# The Role of the Opinion Leader Research Process in Informing Policy Making for Improved Nutrition: Experience and Lessons Learned in Southeast Asia

**DOI:** 10.1093/cdn/nzaa093

**Published:** 2020-05-22

**Authors:** Amy Weissman, Tuan T Nguyen, Hoa T Nguyen, Roger Mathisen

**Affiliations:** 1 FHI 360, Asia Pacific Regional Office, Bangkok, Thailand; 2 Alive & Thrive Southeast Asia, FHI 360, Hanoi, Vietnam; 3 Institute for Families in Society, University of South Carolina, Columbia, SC, USA

**Keywords:** opinion leader, policy advocacy, policy making, nutrition, Southeast Asia, breastfeeding

## Abstract

Opinion leader research (OLR) has been widely used in public health to identify influential persons or organizations to affect health care practice, inform policy-making processes, and help shape communication strategies. We used OLR to gather information related to barriers and possible solutions to guide strategic engagement for strengthening policy making for improved maternal, infant, and young child nutrition (MIYCN) practices in 5 Southeast Asian countries—Cambodia, Laos, Indonesia, Timor-Leste, and Thailand. In most countries, MIYCN policies and policymaker interest exist, but effective implementation and/or enforcement of current policies is weak. This article aims to share our experience in and lessons learned from using OLR as an advocacy tool: It helped identify opinion leaders with interest and influence to affect nutrition-related policies, it raised opinion leaders’ interest in MIYCN, and it identified themes that would help generate political priority setting. Based on our experience, we recommend OLR as a strategic activity for informing and generating support for MIYCN policy-making processes.

## Teaser

Lessons from using opinion leader research (OLR) to improve maternal, infant and young child nutrition (MIYCN) policy-making process in Southeast Asia are shared.

## Introduction

Opinion leaders are individuals with social influence within groups who typically serve as the hub of an interpersonal communications network (1). Because they are considered credible and trustworthy, these leaders typically are role models for others, and their opinions and behaviors are well respected. This means opinion leaders can support the adoption of new ideas or actions, particularly at an accelerated pace (2). Leveraging opinion leaders requires identifying them and understanding their prevailing perceptions and where and how they transition from first knowing of an innovation to forming an attitude toward it, deciding to adopt or reject it, and implementing and confirming it (1–3).

The opinion leader approach has been used in business and marketing, political science, sociology and psychology, education, and public health (3). In low-, middle- and high-income countries throughout the world, opinion leaders have helped promote evidence-based health care practice and health promotion and disease prevention (3). According to a systematic review of 18 randomized controlled trials involving ∼300 hospitals in the United States, Canada, China (Hong Kong), Argentina, and Uruguay across 63 outcomes, the use of local opinion leaders was associated with an increase in compliance with desired clinical practices for better health care outcomes (4). Furthermore, this approach has been used to investigate e-cigarette use in school adolescents in France (5), identify influential and credible persons to support the placement of child health promotion on the national agenda of Sweden (6), improve HIV programming in China by helping reduce HIV stigma in health care practice (7), and support HIV prevention in India (8).

However, apart from the work of Alive & Thrive—a global initiative to save lives, prevent illness, and ensure healthy growth and development through optimal maternal nutrition, breastfeeding, and complementary feeding practices (9)—peer-reviewed papers documenting the use of the opinion leader approach to promote maternal, infant, and young child nutrition (MIYCN) are limited. Yet, improved breastfeeding is critical to optimal MIYCN and child development (10, 11), and country-specific advocacy that strengthens MIYCN policies can effectively increase breastfeeding practices (12, 13). Of note, because MIYCN is inclusive of infant and young child feeding (IYCF), in this article we use the term MIYCN despite IYCF being the common term used when the opinion leader research (OLR) was conducted.

In Bangladesh, Ethiopia, and Vietnam, Alive & Thrive identified opinion leaders who had significant influence in MIYCN policies, programming, and investment and who spanned political, cultural, and health realms (13). The OLR aimed to understand these opinion leaders’ awareness of nutrition and their ideas for MIYCN advocacy goals and strategies, including appropriate messengers and messages, communication channels, and points of engagement (13). Findings from the OLR provided insights on these leaders’ perceptions and priorities and led to the identification of advocacy targets and partners, tailored messages and materials, and preferred communication channels (13).

The OLR experience in these countries served as a model for replication in other countries in Southeast Asia of varied income level, including Cambodia (low-income); Laos, Indonesia, and Timor-Leste (lower-middle-income), and Thailand (upper-middle-income) (14). These countries were the focus of a second phase of Alive & Thrive funding in Southeast Asia because they were experiencing fast economic growth but persistent malnutrition (15) and breastfeeding rates were at risk of declining (14). Although the overall goal of Alive & Thrive in the second phase was to strengthen infant and young child feeding policies and health systems in Southeast Asia over a 3-y period (2013/2014–2016) (15), 2 of its specific objectives were to strengthen national maternity protection policies, such as paid maternity leave, in Indonesia, Thailand, and Timor-Leste; and to improve breastfeeding practices in Cambodia, Laos, and Timor-Leste (15). More information about the context of these countries is described in another article (14).

This article aims to share the experience in and lessons learned from using OLR as an advocacy tool for gathering information related to barriers and possible solutions to guide strategic engagement for strengthening policy making for improved MIYCN practices in these 5 countries and regionally.

## Methods

During 2015 and 2016, Alive & Thrive, in partnership with UNICEF and in-country research agency, conducted an exploratory, qualitative study using OLR in Cambodia, Laos, Thailand, Indonesia, and Timor-Leste. This study aimed to gather data to help build political and public support and to inform advocacy strategies for enhancing MIYCN policies that would result in improved practices (e.g., early and exclusive breastfeeding and uptake of maternity leave). Across the 5 countries, there were 4 study objectives: *1*) review current policies, programs, and models for MIYCN; *2*) identify barriers and potential solutions to political and public support for MIYCN, particularly breastfeeding; *3*) explore different stakeholders’ motivations for supporting MIYCN, particularly breastfeeding; and *4*) identify channels of communication and points of engagement with opinion leaders.

To meet the first objective—to understand current policies relevant to breastfeeding and maternity protections and current MIYCN practices—in each country, researchers from a contracted local agency reviewed national policy documents and data from reports such Multiple Indicator Cluster Surveys, Demographic and Health Surveys, Nutrition Surveillance, Global Nutrition Report, and the Network for Global Monitoring and Support for Implementation of the International Code of Marketing of Breast-milk Substitutes.

For the remaining objectives, in-country researchers conducted in-depth interviews with purposefully selected participants who were engaged in or who influenced MIYCN policy making or programming. Participants—or advocacy targets—were identified in multiple ways. First, the researchers consulted advocacy partners, considered defenders of the desired aims and policies, for names of opinion leaders. They also reviewed other formative work conducted in these countries, such as legal and law-making reviews and media scans (16), and compared the lists of identified opinion leaders. In-country researchers then compared lists across countries to ensure no category was missed in any site. They also conducted a stakeholder mapping to place stakeholders according to their respective power and interest in the topic as per Ackermann and Eden's framework (17). The framework is a grid of 4 quadrants, with the bottom left corner being low interest and low power and the upper right grid being high interest and high power. Finally, researchers employed a snowball technique during the OLR data collection process whereby a study participant referred other participants (18).

Study participants were representatives of government institutions, national assemblies, parliaments, international nongovernmental organizations, national nongovernmental organizations and civil society, bilateral and multilateral organizations, health workers’ and medical associations, private employers and labor unions, mass media, and breast milk substitute (BMS) companies (**Table 1**). Representatives from government institutions were drawn from a wide variety of ministries (e.g., health, agriculture, and labor) and offices of presidents and vice presidents. Participants from national assemblies and parliaments included representatives from parliamentary committees, institutes, and offices. In Indonesia, representatives from the BMS industry also participated in the opinion leader interviews because they were identified during the participant recruitment process as being relevant to the study.

**TABLE 1:** Number of participants by category and country

	Cambodia	Indonesia	Laos	Thailand	Timor-Leste
Total participants, *n*	25	48	51	12	35
Participants by category, *n*					
Government institutions and/or national assemblies,parliaments	10	17	38	7	9
Development partners (bilateral, multilateral,international, and national nongovernmental organizations and civil society)	12	10	4	1	26
Health workers and medical associations		14	4		
Private employers and labor unions		3		4	
Mass media	3	2	5		
Breast milk substitute companies		2			

Question guides were tailored to each country, to reflect their respective situation, but all covered the following topics: awareness and perceptions of and suggestions for improvement related to MIYCN as a priority; existing nutrition-related policies and their implementation status, monitoring practices, and areas for improvement; the country's policy-making process and funding allocation decision making; and appropriate messages, channels, and key influencers. Questions were framed to help participants identify key barriers and possible solutions to MIYCN policy and practice improvement.

Following informed consent procedures, the in-depth interviews were audio-recorded, except in a few instances when participants refused; in-country researchers also took detailed notes. Recordings from all countries were transcribed and translated into English and then coded in-country and reviewed by the Alive & Thrive research team. In each country, content analysis was conducted by hand based on pre-identified themes to assess commonly held and divergent perspectives. Coded data were reviewed and synthesized by key themes, and in-country researchers examined the frequency of respondents’ common or divergent opinions. For the purpose of this article, the Alive & Thrive research team synthesized the overall findings from the 5 countries to identify key emergent themes. The research protocol was reviewed by FHI 360’s Office of International Research Ethics and national ethics committees for each participating country prior to study implementation.

## Results

Across the 5 countries, trained researchers conducted 171 in-depth interviews with identified opinion leaders (Cambodia, 25; Indonesia, 48; Laos, 51; Thailand, 12; and Timor-Leste, 35) (Table 1) to learn of perceived barriers and possible solutions for strengthening MIYCN policy making (**Table 2**).

**TABLE 2:** Barriers and potential solutions identified by country^1^

	Cambodia	Indonesia	Laos	Thailand	Timor-Leste
Barriers identified					
Lack of policymaker interest		X			
Insufficient breastfeeding policy	X				X
Weak policy implementation, coordination	X	X	X		X
Lack of interventions, particularly SBCC	X				X
Influence of companies on health workers’ practices	X	X	X		
Limited knowledge among mothers and families	X	X	X		
Influence of traditional social practices			X		
Limited knowledge among health workers		X	X		
Workplaces do not support working mothers to breastfeed	X	X			X
Insufficient maternity protection policy				X	
Recommendations					
Education, SBCC on MIYCN		X		X	
Monitor conflict of interest among health workers		X			
Strengthen policy implementation, coordination, andenforcement	X	X	X	X	
Strengthen relevant policies	X		X	X	X
Design more effective MIYCN interventions					X
Strengthen behavior change efforts	X				X

1 MIYCN, maternal, infant, and young child nutrition; SBCC, social and behavior change communications.

The most cited barrier was weak policy implementation and coordination, particularly related to the Code (4 countries). For instance, in Cambodia, some participants mentioned that Cambodia has sufficient policies and strategies to support and promote breastfeeding, but that it needs to implement existing laws and policies and monitor and enforce them effectively. Participants from 3 countries cited the influence of BMS companies on health workers’ practices and the country's policy making, limited knowledge among mothers and families, the influence of traditional social practices, and that workplaces do not support working mothers to breastfeed. For instance, in Indonesia, several participants highlighted the influence of BMS companies and their effort to combine forces when a potential policy is particularly at odds with their revenue potential. Participants in 2 countries cited insufficient breastfeeding policy, such as paid maternity leave; lack of interventions, particularly social and behavior change communications; and limited knowledge among health workers.

To overcome the identified barriers, participants recommended specific measures (Table 2). The most common across all sites was to strengthen relevant policies as well as policy implementation, coordination, and enforcement (4 countries each). For instance, in Thailand, most participants recommended improving maternity protection support in workplaces, such as by establishing requirements for breastfeeding rooms because this would provide women who returned to work the space to express milk or directly feed their infants. Other recommended actions related to providing and/or strengthening MIYCN-related education or social and behavior change communications (2 countries each). In Laos, several participants identified the importance of educating mothers and families about the country's laws and policies that provide entitlements in support of breastfeeding.

## Discussion

As anticipated, our results confirm that OLR is a useful approach for informing MIYCN-related advocacy processes as per the original 3 OLR studies supported by Alive & Thrive in Bangladesh, Ethiopia, and Vietnam; the results also expand our understanding of OLR as part of the policy advocacy process. Based on our experience in Vietnam and in the 5 countries that were the focus of this study, we learned valuable lessons, including that the OLR results are not necessarily as important as the OLR process. We identified these lessons through a series of dialogues they facilitated among the Alive & Thrive research team using a question guide that sought to ascertain high-level recommendations and experience from employing OLR.

First, OLR helps identify opinion leaders with interest and influence to affect policy making, yet from our experience, by interviewing them, influential individuals who were previously uninterested in MIYCN were now interested and engaged. This suggests that the OLR process can help move opinion leaders of influence into Ackermann and Eden's “key player quadrant” (17), thus making OLR a strategic advocacy activity. For instance, national assembly members or parliamentarians and members of the office of the president or vice president are often highly influential but have low interest (bottom left quadrant). However, we found that the OLR process and related advocacy activities shifted them to become critical stakeholders in the policy-making process. This can be seen by several OLR participants officially joining country delegations for subsequent Alive & Thrive- and UNICEF-facilitated biennial regional advocacy meetings, which were held to track MIYCN policy-making progress and update country advocacy strategies. Our experience may be anecdotal; thus, more research in this area is needed.

Second, the in-country researchers and organizations who conducted the OLR were critically important and may have been as influential following the study as the research findings. For instance, in Laos, the National Institute of Public Health, a government agency, led the OLR. This helped increase access to key opinion leaders and created a new champion within the government system. It also helped build commitment to the needed policies (19). Similarly, choosing a lead researcher who was known, respected, and had access to the desired participants was crucial. Doing so helped the OLR build opinion leader trust and engagement. Some of the researchers also disseminated the findings in-country, and all in-country research teams presented at a regional meeting in Bangkok in 2016 with participation from these country stakeholders and other Association of Southeast Asian Nations member states. OLR also positioned the subsequent work by having a key opinion leader who was knowledgeable about the issues and known by those to be engaged in the policy process. For instance, in Timor-Leste, Alive & Thrive hired the OLR researcher to implement the advocacy plan developed based on the OLR. Our experience relates to “researcher positionality”—the researcher being internal or external to the study population. Although the benefits of using insider researchers seem to outweigh any risks (20, 21), further research on insider researchers conducting OLR is needed.

Third, OLR methods can help to understand the enabling environment, which as highlighted in the 2013 *Lancet* series encompasses 3 factors: *1*) knowledge and evidence, which provide context-specific framing of an issue; *2*) politics and governance among a wide variety of stakeholders who have or should have a vested interest in nutrition; and *3*) capacity and resources in nutrition and in alliance building and networking, communication and collaboration, and the leveraging of resources (22). In our experience, OLR helped establish an environment by identifying *ideas—*the ways those involved with an issue understand and portray it—and the inputs to frame the issue to generate political priority and motivate change in that context (23). For instance, the OLR in Vietnam identified child rights as the framing for improved MIYCN policies because the Government of Vietnam is very proud that Vietnam was the second country in the world and first country in Asia to sign the Convention on the Rights of the Child and has publicly stated its commitment to child education, nutrition, health, and well-being. Through the OLR, human capital development was identified to resonate well with policy makers in Indonesia, whereas economic and scientific arguments were indicated in the other countries.

While reviewing our experience, it is important to recognize that the purpose of this article is to share experience in and lessons learned from conducting OLR in 5 Southeast Asian countries and that these lessons were identified by the primary Alive & Thrive-led research team—and not with input from in-country research teams. This article was not intended to provide a thorough analysis of the 5 country's results by participant subgroup; thus, stakeholders’ opinions and findings across different countries and across the region may not be generalizable. Notwithstanding, other studies, 1 of which was conducted in Cambodia, have identified similar themes (13, 24, 25). Although the information presented is limited, we believe this article offers some important insights for using OLR to inform policy making for improved nutrition in Southeast Asia.

In conclusion, as in the original 3 countries, OLR was found to be a strategic advocacy activity in the 5 replication countries that was instrumental in engaging potential opinion leaders and tapping into their influence, interest, function, and agenda. It was also critical for identifying how to frame the issues to facilitate a commitment for change. Given our experience, we recommend employing OLR to build the political and social support needed to improve MIYCN policies and practices, particularly breastfeeding and maternity protection.

## Notes

Author disclosures: The authors report no conflicts of interest.

Abbreviations used: BMS, breast milk substitute; IYCF, infant and young child feeding; MIYCN, maternal, infant, and young child nutrition; OLR, opinion leader research.
